# DNA/MVA Vaccines for HIV/AIDS

**DOI:** 10.3390/vaccines2010160

**Published:** 2014-02-28

**Authors:** Smita S. Iyer, Rama R. Amara

**Affiliations:** Emory Vaccine Center, Division of Microbiology and Immunology, Yerkes National Primate Research Center, Emory University, Atlanta, GA 30329, USA

**Keywords:** adjuvant, SIV, rhesus macaque, CD40L, GM-CSF

## Abstract

Since the initial proof-of-concept studies examining the ability of antigen-encoded plasmid DNA to serve as an immunogen, DNA vaccines have evolved as a clinically safe and effective platform for priming HIV-specific cellular and humoral responses in heterologous “prime-boost” vaccination regimens. Direct injection of plasmid DNA into the muscle induces T- and B-cell responses against foreign antigens. However, the insufficient magnitude of this response has led to the development of approaches for enhancing the immunogenicity of DNA vaccines. The last two decades have seen significant progress in the DNA-based vaccine platform with optimized plasmid constructs, improved delivery methods, such as electroporation, the use of molecular adjuvants and novel strategies combining DNA with viral vectors and subunit proteins. These innovations are paving the way for the clinical application of DNA-based HIV vaccines. Here, we review preclinical studies on the DNA-prime/modified vaccinia Ankara (MVA)-boost vaccine modality for HIV. There is a great deal of interest in enhancing the immunogenicity of DNA by engineering DNA vaccines to co-express immune modulatory adjuvants. Some of these adjuvants have demonstrated encouraging results in preclinical and clinical studies, and these data will be examined, as well.

## 1. Introduction

The RV144 recombinant canary pox vector, ALVAC/gp120 vaccine efficacy trial was the first to demonstrate a reduction in the risk of HIV acquisition by an HIV vaccine [1]. This vaccine-mediated efficacy, although moderate and appearing to wane over time, has reinvigorated the HIV vaccine field and renewed confidence towards the development of an effective HIV vaccine. There is a lot more work that needs to be done to develop an efficacious HIV vaccine, and the recently reported failure of the HIV Vaccine Trials Network (HVTN) 505 DNA/adenovirus 5 vaccine is a sobering reminder of the challenges we face towards realizing this goal [2].

**Figure 1:**
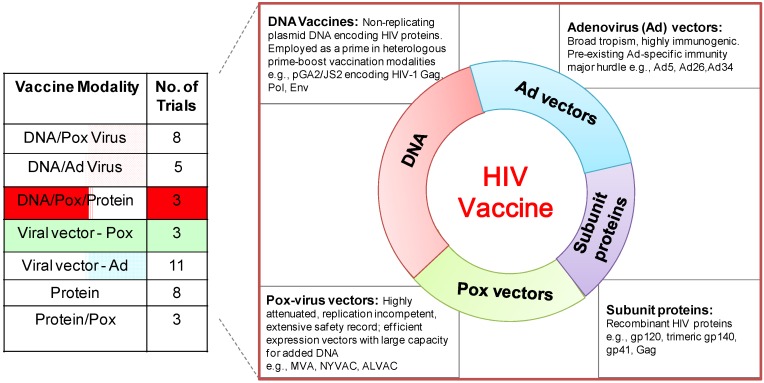
Phase I/II clinical trials (ongoing/scheduled) of HIV vaccines. (Left) the table shows Phase I/II HIV vaccine trials by vaccine modality obtained from the International AIDS Vaccine Initiative (IAVI) database of vaccine candidates in clinical trials [4].

Post RV144, at least two strategies of vaccine development can be identified. The first consists of building upon the poxvirus prime, subunit protein boost employed in RV144 with the goal of enhancing immunogenicity and increasing efficacy, and the second involves pursuing diverse vaccine regimens to identify more effective vaccine strategies [3]. Currently, the main types of vaccines being developed in the clinic for HIV use recombinant protein subunit vaccines, such as the glycoprotein (gp) 120-protein fragment of the HIV envelope tested in the RV144 study, recombinant virus-vectored vaccines, such as ALVAC, New York Vaccinia Virus (NYVAC), modified vaccinia Ankara (MVA) and adenovirus serotypes, and DNA vaccines, typically used to prime immune responses in heterologous prime-boost vaccine modalities. Vaccine modalities based on DNA prime comprise a significant fraction of the current scheduled or ongoing Phase I and II HIV vaccine trials across the world (Figure 1). While plasmid DNA has demonstrated limited efficacy as a stand-alone vaccine, DNA in combination with viral vectors/protein shows a striking synergy in immune responses compared to either component alone. Innovations in the DNA platform with improved DNA delivery methods, such as electroporation and adjuvanting DNA with immunomodulatory molecules, have enhanced DNA immunogenicity. Here, we will briefly review the immunogenicity and efficacy studies using DNA as a prime in non-human primates and focus on pre-clinical and clinical studies of DNA/MVA HIV vaccines.

## 2. A Brief History of DNA Vaccines

**Figure 2:**
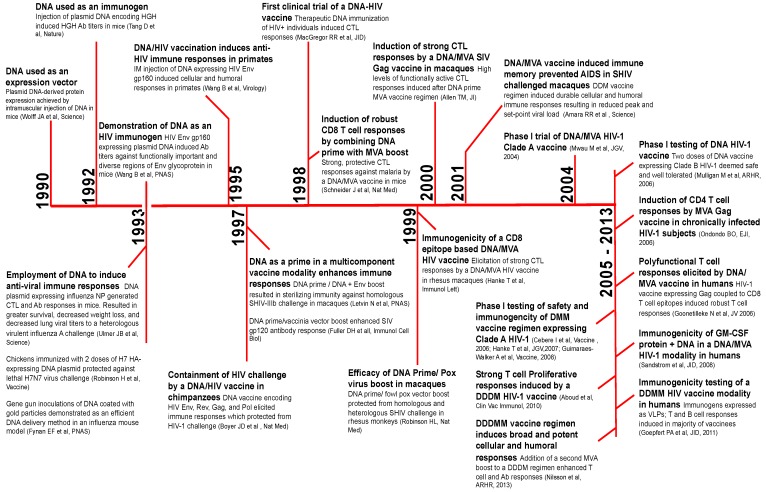
The timeline of benchmark studies resulting in the development of DNA as a prime for the DNA/MVA HIV vaccine modality. The timeline of key studies resulting in the clinical application of DNA as an immunogen for DNA/MVA HIV vaccines. HGH, human growth hormone; NP, nucleoprotein; CTL, cytotoxic T-cell; Ab, antibody; HA, hemagglutinin; Env, envelope; IM, intramuscular; GM-CSF, granulocyte macrophage colony stimulating factor; SIV, Simian immunodeficiency virus; SHIV, Simian/Human immunodeficiency virus.

In this section, we provide a brief timeline of benchmark studies leading to the development of DNA/MVA HIV vaccines beginning with the use of plasmid DNA to induce immunity against influenza more than two decades ago (Figure 2). The first use of naked plasmid DNA as an expression vector was demonstrated in 1990 [5]. In 1992, Tang *et al*. employed DNA as a simple means to elicit immune responses against non-self antigens [6]. Mice were immunized intradermally with plasmid encoding human growth hormone (HGH) using a gene gun approach. Remarkably, DNA immunization induced serum HGH Ab (antibody) responses, which were augmented by a booster shot. This simple and unique technique of genetic immunization generated considerable excitement for two reasons; first, DNA immunization would overcome the time-consuming need for protein purification necessary for protein immunizations; and second, DNA encoding viral proteins could serve as a vaccine against viral infections.

The latter possibility was quickly realized and elegantly demonstrated by Ulmer *et al*. in 1993 [7]. cDNA encoding the influenza A nucleoprotein (NP) was injected intramuscularly at a dose of 100–400 μg at zero, three and six weeks in mice. Immunization with 100 μg NP DNA resulted in NP-specific antibodies and cytotoxic T-cell (CTL) responses, which resulted in protection against a heterologous influenza challenge. Around the same time, Robinson *et al*. demonstrated the efficacy of a hemagglutinin (HA)-expressing DNA plasmid in an avian influenza model [8]. Additional studies in mice demonstrated that gene gun delivery of HA-encoding DNA-coated gold particles to the epidermis conferred superior protection compared to intramuscular, intravenous and intranasal routes of DNA inoculation [9]. Together, these data showed for the first time that the injection of naked DNA encoding a conserved viral protein was a simple, yet effective way to induce CTL responses and high-titer antibodies. DNA-induced immunity was durable and protective, resulting in enhanced protection from a heterologous, highly virulent influenza challenge.

In the same year, Wang *et al*. demonstrated the ability of plasmid DNA to induce anti-HIV immune responses [10]. Immunizations in mice comparing DNA *versus* protein immunogens showed that DNA immunization elicited higher titers of antibody against functionally important, diverse regions of HIV Env, V3 loop, immunodominant and fusogenic regions of gp41 and the conformational CD4 binding site. Subsequent studies by Peet *et al*. also showed higher V3 antibody titers in response to DNA compared to protein immunization [11]. The qualitative differences in antibody responses to DNA *versus* protein immunogens could be attributed to the expression of viral proteins in conformations that mimic natural infection in DNA-transfected cells. This could result in the effective presentation of important epitopes to the immune system. Indeed, it is this feature of DNA that makes it highly desirable as an immunogen.

In all, the data showed that DNA was immunogenic in mice. However, data in primates was lacking. Previous studies had shown that non-human primate (NHP) muscle had the ability to take up injected plasmid DNA, albeit at a lower level than mice [12]. Comparison of plasmid DNA expression in mice *versus* monkey muscle demonstrated about 30-fold lower expression in monkeys compared to mice. While differences in body size and/or muscle histology across species could contribute, it raised questions about the potential immunogenicity of DNA in humans. This concept was formally tested in primates: cynomolgus monkeys were inoculated with 100 μg of plasmid DNA expressing HIV gp160 protein [13]. After four DNA immunizations, humoral and cellular responses were induced, indicating that DNA was immunogenic in primates and would likely be immunogenic in humans. The immunogenicity raised by DNA was shown to be protective; Boyer *et al*. demonstrated DNA vaccination protected chimpanzees from acquisition of a highly attenuated, heterologous HIV-1 challenge [14]. These data together with other seminal studies in the field, described later in this review, paved the way for the development of DNA/MVA HIV vaccines [15,16,17,18,19,20,21,22,23,24,25,26,27,28,29,30,31,32,33].

## 3. How Is DNA Immunogenic?

The mechanisms by which intramuscular DNA injection primes an immune response to encoded foreign antigens is the subject of intense discussion for two main reasons: first, the muscle has relatively fewer professional antigen presenting cells (APC) [34,35]. Unlike the skin, which contains Langerhans dendritic cells (DC), the muscle has low frequencies of DCs and macrophages. Indeed, intradermal DNA immunization raises higher magnitude immune responses compared to intramuscular DNA vaccination [22]. Second, myocytes are not professional APCs and, in general, express low levels of major histocompatibility (MHC) Class I and co-stimulatory molecules, and this raises questions as to their effectiveness in priming CD8 T-cells. As a result, there has been a great deal of interest in dissecting the mechanisms involved and key cellular players in DNA immunization, and numerous studies have assessed the relative contribution of myocytes *versus* APCs in priming CTL responses after DNA immunization [35,36,37].

The current paradigm is that the immunogenicity of DNA vaccines results from the cooperative action of three possible mechanisms of antigen presentation: (1) transfected myocytes presenting antigen to CD8 T-cells directly; (2) cross-priming by professional APCs, such as DCs, by the transfer of antigen from transfected myocytes; and (3) direct transfection of APCs [35,36]. Scenarios 2 and 3 seem to be dominant mechanisms among the three. Presentation of antigen to T-cells in Scenarios 2 and 3 would require the migration of antigen-loaded APCs from the site of vaccination to the T-cell zone of the draining lymph node for presentation to T-cells. This would be especially critical in the context of a DNA vaccine encoding cell-associated antigen. However, for secreted antigens, e.g., in the form of virus-like particles (VLPs), one can envision the diffusion of VLPs to the secondary lymphoid organs.

The relative contribution of somatic cells *versus* APCs in antigen presentation would depend on the DNA formulation, method of administration, promoter driving antigen expression in DNA plasmid, the form of DNA expressed antigen (secreted, cell-associated) and other attributes of the host [37,38,39]. For instance, co-injection of adjuvants either expressed by plasmid DNA or in the vaccine formulation could improve the magnitude of immune responses by augmenting the recruitment of APCs to the site of vaccination and enhancing co-stimulatory capacity. Delivery of DNA by electroporation also induces significantly stronger immune responses, in part by increasing the transfection efficiency of myocytes (and, potentially, APCs) by about 100-fold [40]. An understanding of the mechanisms by which plasmid DNA primes immune responses is critical to the design of more immunogenic and effective DNA vaccines. As will be discussed in subsequent sections, multiple means have been employed to improve immune responses primed by DNA vaccines, and these studies have contributed to our understanding of key characteristics facilitating effective immune priming.

### 3.1. DNA As a Prime for an HIV Vaccine

The ability of naked DNA encoding antigen to induce T- and B-cell responses is remarkable in its simplicity and relative safety. DNA, however, is poorly immunogenic, and the magnitude of immune response is typically low [15,40]. Indeed, antigen load realized by DNA immunization limits immune responses. Therefore, there was a great deal of interest in employing DNA delivery methods that would overcome this limitation. Improved DNA delivery methods exist, such as transfection agent bupivacaine administered either in conjunction with or prior to DNA immunization [15,41], gene gun immunization to deliver DNA-coated gold micro-particles in the skin [9,17] and the use of electroporating agents to enhance DNA immunogenicity [40,42,43]. Because immunity conferred by DNA vaccines is durable and can be boosted by many heterologous vaccine vectors, strategies to boost immune responses primed by DNA have been remarkably successful.

Letvin *et al*. evaluated the immunogenicity and efficacy of DNA vaccine with and without a protein boost in primates [16]. Monkeys received three shots of 1–2 mg plasmid DNA encoding HIV gp160 Env gene from the HXBc2 clone of HIV IIIB, followed by boosting with DNA + Env protein from the parental HIV IIIB strain. This vaccination regimen elicited high antibody titers against gp120, which played a role in complete protection from an intravenous challenge with a simian/human immunodeficiency virus (SHIV) HxB2 challenge, suggesting that this immunization regimen induced sterilizing immunity against HIV. While the challenge model was not rigorous due to the fact that SHIV HxB2 replicates at low levels and uses an Env gene derived from a neutralization-sensitive T-cell line culture laboratory strain of HIV, this immunization strategy provided the proof-of-concept that DNA-primed antibody responses could be augmented by a protein boost.

Soon thereafter, Robinson *et al*. demonstrated that boosting with a live viral vector was superior to protein boosting [19]. The data showed a synergy between DNA prime and poxvirus vector boosting, which enhanced both antibody and cellular immune responses. A comparison of intradermal or gene gun delivered DNA prime followed by protein or fowlpox virus boost expressing Gag, Pol, Env, and Nef genes of SHIV-IIIb showed that intradermal DNA priming followed by fowlpox boosting protected 3/4 of animals from two consecutives homologous SHIV–IIIb challenges. Two thirds of protected animals contained a subsequent highly pathogenic SHIV 89.6P challenge. This study demonstrated that boosting DNA primed responses with a live, replication defective viral vector augments humoral immune responses. Indeed, a prior study by Fuller *et al*. demonstrated that single inoculation of recombinant vaccinia vector expressing gp160 resulted in a striking enhancement in gp120 specific IgG titers primed by DNA, compared to boosting with DNA [17]. These data indicated that recombinant vaccinia vectors could synergize with DNA to enhance immune responses.

### 3.2. Development of DNA Prime/MVA Boost Vaccine

MVA is a highly attenuated virus derived from the vaccinia virus strain, Ankara [44]. Its ability to infect multiple cell types, including professional APCs, and inability to initiate a second round of replication in mammalian cells makes it a safe and immunogenic expression vector. More so, MVA is safe even in immune compromised individuals. As a vector, MVA has many desirable properties. It has a large capacity for added DNA and is a highly efficient expression system; the latter is due to the fact that in infected cells, the block in virus assembly occurs after DNA replication and protein synthesis [45]. Several studies showed that intramuscular injection of recombinant MVA expressing foreign genes elicits strong cellular and humoral responses [46,47].

In 2001, Drs. Robinson and Amara reported that immune memory established by a DNA/MVA vaccine consisting of two DNA primes and a single MVA boost (DNA DNA MVA; DDM) vaccine regimen blunts acute viremia and rapidly controls set point viremia to below the level of detection and provides long-term viral control of a high-dose pathogenic SHIV 89.6P intrarectal infection in rhesus macaques [22]. The vaccine did not protect from mucosal SHIV 89.6P challenge, but reduced peak and set-point viremia, protected from CD4 T-cell loss and prolonged survival [48]. Subsequent studies comparing a Gag-Pol-Env vaccine to a Gag-Pol vaccine demonstrated better viral control, with the former indicating anti-Env binding Ab together with CD8 T-cells was necessary for containment of virus [49]. Follow up studies demonstrated that long-term viral control was associated with the maintenance of low breadth and low frequency IFNγ, IL-2 co-producing anti-viral T-cells [48]. In two animals, mutational escape of the virus in CD8 Gag epitopes led to ineffective immune control and re-emergence of virus, demonstrating the critical role of vaccine-induced CD8 T-cells in controlling virus [50]. Indeed, in a subsequent study, we documented that transient CD8 depletion in animals with <80 copies of virus per milliliter of plasma resulted in a greater than three-log-fold increase in viral titers, which was controlled after the reemergence of anti-viral CD8 T-cells [51]. Thus, memory responses engendered by a Gag-Pol-Env DDM vaccine resulted in the containment of acute viral infection by rapid recall of memory T- and B-cells. Vaccine induced protection was maintained in 22/24 animals for up to 200 weeks post challenge.

## 4. DNA/MVA *vs*. MVA-Only Vaccines

In an effort to understand the influence of DNA prime on immune responses boosted by MVA and the contribution to protection, we compared immune responses and protection in DNA/MVA (DDM modality) and MVA-only (MMM modality) vaccines [52]. More recently, we also compared the immunogenicity of a DDMM modality with the MMM modality [53]. These studies demonstrated that MVA vaccination following a DNA prime elicits strong CD4 and CD8 T-cell responses. In contrast, priming with MVA (in the absence of DNA) elicits weak CD4 and CD8 T-cell responses, which are not boosted efficiently by repeated MVA immunizations. On the other hand, antibody responses are boosted in both modalities (in the presence or absence of DNA primes), resulting in strong T-cell and antibody responses in the DDMM group and moderate T-cell and strong antibody response in the MMM group.

In the DDM study, we found the evidence for DNA vaccine priming qualitatively different immune responses compared to MMM vaccine. Post SHIV 89.6P challenge, the binding titers against HIV gp140 were comparable between the two groups. Interestingly, however, neutralizing Ab titers against homologous, as well as heterologous Envs were slower to emerge post challenge in the MMM regimen compared to the DDM regimen. Plasma viral load was comparable between vaccine groups, but examination of infected CD4 T-cells by intracellular p27 staining showed a higher frequency of infected cells in the MMM group. Furthermore, slower contraction of both cellular and humoral responses in the MMM group indicated the possibility for sequestered/persistent virus within tissues. Despite these differences, both vaccine regimens achieved similar viral control [52]. These promising data along with other seminal studies in the field shaped the concept of a DNA/MVA heterologous prime-boost vaccine regimen for HIV.

## 5. Adjuvanted DNA Vaccines

In this section, we will review some of the molecular adjuvants used to augment immune responses induced by DNA, discuss known mechanisms of action and examine the efficacy of adjuvanted DNA vaccines in preclinical studies.

Among the first experimentation of adjuvanted DNA was a mouse study showing the augmentation of T- and B-cell responses to hepatitis virus core protein (HCV) by co-immunizing with DNA plasmids encoding HCV antigen and either interleukin-2 (IL-2), interleukin-4 (IL-4) or granulocyte macrophage colony-stimulating factor (GM-CSF) [54]. All adjuvants enhanced HCV seroconversion; 80% of mice receiving adjuvanted HCV DNA made detectable anti-HCV antibodies compared to 40% of mice immunized with HCV DNA alone. Co-immunization with IL-2 DNA resulted in the strongest induction of CD4 T-cell responses as measured by *ex vivo* proliferation of splenocytes in response to rHCV nucleocapsid protein. Furthermore, spontaneous CTL activity in splenocytes was also strikingly enhanced by co-immunizing with IL-2. Effector T-cells derived from mice co-immunized with IL-2 secreted the highest levels of IL-2 and IFN-γ, while IL-4 adjuvanted effectors secreted IL-2 and IL-4, but not IFN-γ. These data indicate that adjuvants can alter not only the magnitude, but also the quality of DNA elicited immune responses.

IL-2 has also shown promise as a DNA adjuvant in preclinical and clinical studies. Studies in non-human primates showed that IL-2 adjuvanted DNA significantly attenuated disease by decreasing set-point viremia in a SHIV 89.6P challenge model [55]. This effect was largely mediated by the induction of strong and durable cytolytic CD8 T-cell responses. In a clinical study, higher T-cell response rates were observed with IL-2 adjuvanted DNA (IL-2 administered as a fusion protein with immunoglobulin), when IL-2 was given 48 h post-vaccination compared to concurrent administration [56]. This could reflect a greater need for IL-2 during early T-cell responses relative to the requirement by innate cells.

There have been a wide range of other molecular adjuvants tested to enhance the immunogenicity of DNA, including IL-12 and IL-15, transcription factors, such as interferon regulatory factors, growth factors and co-stimulatory molecules administered either as soluble forms or via expression vectors [40]. These data demonstrate that: (1) increasing local cytokine production at the site of antigen administration can enhance the immunogenicity of DNA; (2) the type of cytokine adjuvant (T_H_1 *vs*. T_H_2) can influence the quality of the CD4 helper response, which, in turn, can impact humoral responses; (3) chemokines and growth factors that induce the migration of APCs to the immunization site increase DNA immunogenicity; and (4) an ideal adjuvant augments both cellular and humoral immune responses. Thus, adjuvanting DNA vaccines provide a strategy to effectively manipulate the immune response based on the infectious agent and the host. In our laboratory, we have found promising results with two adjuvants, which are discussed in the following sections.

### 5.1. GM-CSF Adjuvanted DNA Vaccine

The capacity of GM-CSF to recruit, induce expansion and stimulate the differentiation of APCs makes it highly desirable as an adjuvant for DNA immunizations [57,58]. An elegant series of experiments by Haddad *et al*. showed that the delivery of pGM-CSF at the same site of immunization was critical to enhance immunogenicity [59]. Using *Plasmodium yoelii* model in mice, they demonstrated that pGM-CSF enhanced immunogenicity and protection by inducing the local influx of APCs, and this effect was not duplicated by injecting pGM-CSF either intravenously or at a distant intramuscular site. Thus, local and paracrine effects of GM-CSF at the site of vaccination appear to be a critical factor in enhancing immunogenicity. This is a clinically important characteristic, as it reduces the chance for off-target effects and resulting toxicity.

Plasmid encoded GM-CSF has been demonstrated to be an effective DNA adjuvant in several DNA/MVA immunization studies. First, we examined GM-CSF adjuvanted DNA in a DDM modality [60]. GM-CSF adjuvant was included only during the DNA primes. Immunogens were derived from the chimeric SHIV isolate, SHIV 89.6, and animals were challenged with a high dose SHIV 89.6P, seven months after the MVA boost. Interestingly, GM-CSF did not significantly enhance the titer of anti-Env antibody, but enhanced the quality of the antibody response. GM-CSF adjuvanted animals demonstrated an earlier peak in neutralizing Ab responses after infection, which resulted in four times lower viral titers at Week 2 post infection. Thus, the GM-CSF adjuvanted DDM vaccine regimen resulted in acute viral containment. In a follow up study using the DMMM vaccination modality, we found that adjuvanting DNA with GM-CSF enhanced the avidity of anti-Env binding antibody that was associated with the enhanced control of peak SHIV 89.6P viremia [61].

In a third study, we tested the efficacy of GM-CSF adjuvanted DNA prime in a DDMM vaccination modality with SIV239 immunogens and repeat, moderate-dose intrarectal challenges with a relatively neutralization sensitive SIVsmE660 virus [62]. Adjuvanting DNA with GM-CSF resulted in the protection of 70% of the vaccinated animals compared to 25% and 0% in unadjuvanted and control groups. Correspondingly, an enhancement in the B-cell response was observed in the GM-CSF adjuvanted group with a higher avidity of anti-Env binding antibody, increased titers of neutralizing and Antibody-dependent cell mediated cytotoxicity (ADCC) activities and increased binding titers of anti-Env IgA in rectal mucosa. The avidity of anti-Env IgG for challenge Env was identified as a strong correlate of protection.

**Figure 3:**
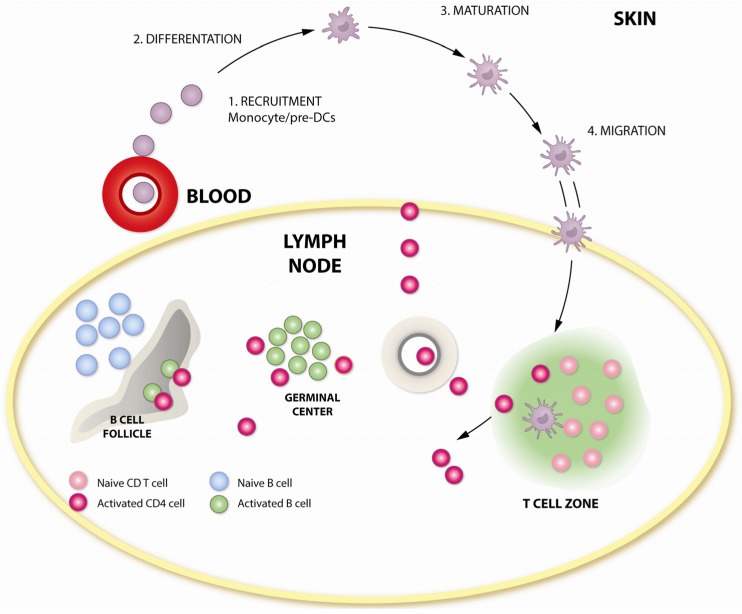
Adjuvant activity of GM-CSF in modulating T- and B-cell responses. GM-CSF influences critical steps in antigen presentation, which could enhance vaccine-induced T-and B-cell responses. GM-CSF increases the recruitment of myeloid progenitor cells, induces their differentiation and maturation, resulting in enhanced Class II expression and antigen presentation. Enhanced migration of activated APCs to lymphoid tissue could enhance vaccine-induced T- and B-cell responses.

In all three studies, protection conferred by DNA adjuvanted with GM-CSF appeared to be mediated by the effects of GM-CSF in the B-cell compartment, as the magnitude of T-cell responses were not significantly enhanced by GM-CSF. It is possible, however, that the quality of the B-cell helper CD4 responses may have been enhanced by GM-CSF, although this was not directly determined. Another possibility was that GM-CSF mediated the enhancement in DC maturation and function, especially that of myeloid DCs, which express receptors for GM-CSF, which could play a role in augmenting the quantity and quality of anti-Env B-cell response. The schematic in Figure 3 outlines the points of action of GM-CSF in adjuvanting immune responses. The GEO-D03 DNA vaccine that co-expresses HIV-1 clade B proteins, Gag, protease, RT, gp160 Env, Tat, Vpu and Rev, as non-infectious VLPs and human GM-CSF [63], has completed a human Phase I study in the U.S.

### 5.2. CD40L Adjuvanted DNA/MVA Vaccines

The co-stimulatory role of CD40L on T-cells and B-cells makes it a highly desirable adjuvant. Pre-clinical studies in our laboratory have shown promising results with CD40L-adjuvanted DNA/MVA vaccines in rhesus macaques (work in progress). CD40L is a type II transmembrane protein of the tumor necrosis factor super family, expressed transiently by activated CD4 T-cells [64]. Its receptor, CD40, is constitutively expressed by numerous cell types, most notably APCs, such as immature DCs and B-cells. Engagement of CD40 on DCs by CD40L induces upregulation MHC-class II and co-stimulatory molecules, secretion of inflammatory cytokines, such as IL-12, and maturation and survival of DCs; factors central to the initiation of a cellular immune response. Ligation of CD40 on DCs is critical for DC licensing in order to prime antigen-specific CD8 T-cells. Several studies have shown that systemic administration of agonistic anti-CD40 antibody results in CD8 T-cell-mediated tumor eradication. These results support a model in which the induction of strong stimulatory signals by CD40L licenses DCs to prime CD8 T-cells [65,66]. 

CD40L also plays an important role in inducing antibody responses by promoting B-cell proliferation and immunoglobulin class switching [67]. In naive B-cells, ligation of CD40 together with B cell receptor (BCR) engagement induces clonal expansion and differentiation to short-lived plasmablasts or into rapidly proliferating germinal center (GC) B-cells [68]. Ligation of CD40 on GC B-cells via CD40L on T_FH_ cells is necessary for affinity maturation and class switching to IgG. Continuous CD40 signaling together with input from the cytokines, IL-21 or IL-4, is required for GC B-cell proliferation, and removal of CD40L results in plasma cell differentiation [69].

The stimulatory functions of CD40L in inducing cellular and humoral immune responses led to many strategies for using it as a vaccine adjuvant. However, systemic administration of CD40L is associated with hepatotoxicity and other side effects [70]. Therefore, local and transient expression of CD40L by means of vaccine expression vectors is more suitable for use of CD40L as a vaccine adjuvant. In mice, co-immunization of plasmid DNA expressing secreted HIV Gag together with an expression vector expressing soluble multimeric form of CD40L resulted in enhancement in CD8 cytolytic responses [71]. Gomez *et al*. demonstrated that soluble hexameric CD40L protein (sCD40L) administered during both DNA prime and NYVAC boosting potentiated both cellular and humoral responses in mice [72]. sCD40L increased the frequency of antigen-specific T-cells by two-fold in the DNA/MVA regimen and by two-fold in the DNA/NYVAC regimen. Adjuvanting with CD40L also decreased the dose of antigen required by 10-fold during the DNA prime. CD40L has also been shown to enhance the immunogenicity of ALVAC HIV vaccines in mice [73].

**Figure 4:**
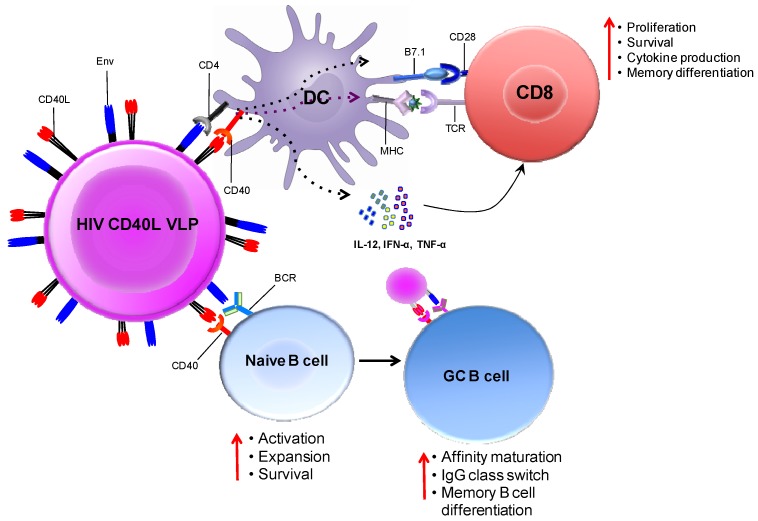
Immune enhancement by CD40L adjuvanted DNA/MVA HIV vaccines. The schematic conceptualizes the mechanisms by which the co-expression of membrane CD40L on virus-like particles (VLPs; produced by DNA transfected cells or MVA infected cells) expressing HIV Env enhances cellular and humoral responses. DCs and B-cells both receive co-stimulatory signals from VLPs by the ligation of trimeric membrane-bound CD40L (on VLP) and CD40 (on DC or B-cells). VLPs bind to DCs via the interaction of gp120 (on VLP) and CD4 (on DC). This interaction is likely to jump-start CD8 T-cell responses by lowering the threshold for DC activation. Second, VLPs can also bind Env-specific B-cells via the interaction between gp120 (on VLP) and the B-cell receptor (surface Ig). Engagement of the BCR together with CD40 ligation results in the activation and differentiation of naive B-cells. In the germinal center, CD40 signaling enhances affinity maturation, class switch recombination and differentiation to memory B-cells. This model predicts that CD40L-delivered co-stimulation signals will enhance T- and B-cell responses to vaccine antigen. Schematic not drawn to scale; VLPs enlarged relative to immune cells for clarity.

In our laboratory, we designed CD40L-adjuvanted DNA/SIV vaccines to co-express macaque CD40L with the native trimeric form of SIV Env on the membrane of the transfected cell/VLP. This expression strategy promotes the multimerization of the ligand, which is critical for its adjuvant activity [74]. Figure 4 conceptualizes the mechanism by which CD40L expressed by soluble VLPs adjuvants T- and B-cell responses *in vivo*. We observed that CD40L adjuvant enhances protection from the acquisition of both neutralization-sensitive (SIVsmE660), as well as -resistant (SIVmac251) intrarectal repeat dose SIV challenges. In addition, the CD40L adjuvanted animals that became infected following SIVmac251 challenge showed better viral control, did not develop AIDS and showed enhanced survival. These results strongly support clinical testing of CD40L adjuvanted DNA/MVA HIV vaccine.

## 6. DNA/MVA HIV Vaccines in Clinical Trials

The immunogenicity and safety of plasmid DNA in animal studies provided the impetus for clinical testing of DNA encoding HIV proteins in humans. Over the years, a number of studies have been performed in HIV uninfected and infected volunteers; these studies have not only contributed to our understanding of immunogenicity and the safety of DNA vaccines in humans, but have also yielded insights into strategies to enhance DNA-induced immune responses. In this section, we review the immunogenicity of selected human studies that have employed DNA either alone or as a prime in a heterologous prime-boost vaccination regimen.

Among the first clinical trials of DNA HIV vaccines was a therapeutic vaccine study by Calarota *et al*. determining the ability of plasmid DNA encoding HIV nef, rev and tat in raising CD8 T-cell responses in symptom-free HIV-infected patients [75]. The vaccine regimen consisted of three DNA immunizations over a six-month period. Interestingly, even a low dose of 100 µg of DNA induced CTL responses in eight out of nine immunized patients. Although responses were transient and no decrease in viral load occurred, the study provided the proof-of-principle that immunization with DNA could induce CD8 T-cell responses in HIV infected patients. The results suggested that a higher dose of DNA could yield more robust T-cell responses. This strategy was tested in a dose-escalation trial by MacGregor *et al*., where HIV-individuals were immunized with 100, 300 or 1,000 µg of DNA encoding Env and Rev of HIV-1 MN isolate [41]. Fairly robust T-cell responses were induced at a 1-mg DNA dose with an estimate of 0.1% of CD4s responding. While no antibody responses to Env were observed, the potential ability of DNA to prime a T_H_1 CD4 response indicated that combining DNA vaccine with vectors that express antigens at higher levels could enhance T- and B-cell immune responses. Studies by Mulligan *et al*. demonstrated that immunization with DNA expressing HIV antigens was safe and well tolerated in HIV negative individuals and DNA vaccination predominantly induced CD4 T-cell responses [24].

Combining DNA prime with MVA boost engendered a striking T-cell response. Goonetilleke compared the immunogenicity of two DNA primes followed by a single MVA boost (DDM) to two MVA boosts (MM) [26]. While no T-cell responses were observed after the DNA primes, boosting with MVA significantly augmented T-cell responses primed by DNA, resulting in a striking increase in IFNγ^+^ antigen-specific T-cells. On the other hand, human volunteers in the MM modality showed no detectable increase in antigen-specific T-cells. Subsequently, a study by Goepfert *et al*. showed that two DNA primes followed by two MVA boosts induced vigorous, polyfunctional, broad and durable T-cell responses in 90% of vaccinated individuals [32]. Numerous clinical studies have shown that responses primed by DNA are significantly boosted using replication deficient viral vectors [27,76,77,78] and over multiple studies, the following unifying themes emerge.

Foremost, DNA vaccination is safe and well tolerated in most individuals, and no clinical trial to-date has reported the integration of plasmid DNA with the host chromosome. Second, immune responses after DNA priming, despite being low to non-detectable, are induced and significantly amplified by subsequent heterologous boosts with viral vectors. A single DNA prime is insufficient, and >2 DNA primes do not increase immune responses over two DNA primes. Third, intramuscular DNA immunization primes T_H_1 CD4 T-cell responses; replication-deficient viral vectors can significantly enhance T- and B-cell responses primed by DNA. Fourth, combining a DNA prime and a viral vector-boost modality with protein immunization may result in the induction of potent and persistent T- and B-cell responses.

## 7. Conclusions and Perspectives

Since the inception of DNA as a tool to induce immune responses two decades ago, continued advancements and innovations in this vaccine platform are paving the way for its clinical use. The strength of plasmid DNA as an immunogen lies in its ability to mimic natural infection; DNA transfected cells express viral proteins in “natural” conformations, which could result in the effective presentation of important epitopes to the immune system. DNA is excellent at priming both T- and B-cell responses and is especially good at inducing durable CD4 T-cell responses. While the magnitude of these responses is typically low, the use of adjuvanted DNA, electroporation and/or boosting with heterologous vectors represent attractive strategies to augment DNA-primed immune responses. The way forward lies in the understanding of the types of protective immune responses primed by DNA and identifying strategies to harness them to build better vaccines.

## References

[1] Rerks-Ngarm S., Pitisuttithum P., Nitayaphan S., Kaewkungwal J., Chiu J., Paris R., Premsri N., Namwat C., de Souza M., Adams E. (2009). Vaccination with ALVAC and AIDSVAX to prevent HIV-1 infection in Thailand. N. Engl. J. Med..

[2] Hammer S.M., Sobieszczyk M.E., Janes H., Karuna S.T., Mulligan M.J., Grove D., Koblin B.A., Buchbinder S.P., Keefer M.C., Tomaras G.D. (2013). Efficacy trial of a DNA/rAd5 HIV-1 preventive vaccine. N. Engl. J. Med..

[3] Michael N.L. (2012). Rare serotype adenoviral vectors for HIV vaccine development. J. Clin. Invest..

[4] IAVI Report (2013). Clinical Trials Database. http://www.iavireport.org/Trials-Database/Pages/default.aspx.

[5] Wolff J.A., Malone R.W., Williams P., Chong W., Acsadi G., Jani A., Felgner P.L. (1990). Direct gene transfer into mouse muscle *in vivo*. Science.

[6] Tang D.C., deVit M., Johnston S.A. (1992). Genetic immunization is a simple method for eliciting an immune response. Nature.

[7] Ulmer J.B., Donnelly J.J., Parker S.E., Rhodes G.H., Felgner P.L., Dwarki V.J., Gromkowski S.H., Deck R.R., DeWitt C.M., Feredman A. (1993). Heterologous protection against influenza by injection of DNA encoding a viral protein. Science.

[8] Robinson H.L., Hunt L.A., Webster R.G. (1993). Protection against a lethal influenza virus challenge by immunization with a haemagglutinin-expressing plasmid DNA. Vaccine.

[9] Fynan E.F., Webster R.G., Fuller D.H., Haynes J.R., Santoro J.C., Robinson H.L. (1993). DNA vaccines: Protective immunizations by parenteral, mucosal, and gene-gun inoculations. Proc. Natl. Acad. Sci. USA.

[10] Wang B., Ugen K.E., Srikantan V., Agadjanyan M.G., Dang K., Refaeli Y., Sato A.I., Boyer J., Williams W.V., Weiner D.B. (1993). Gene inoculation generates immune responses against human immunodeficiency virus type 1. Proc. Natl. Acad. Sci. USA.

[11] Peet N.M., McKeating J.A., Ramos B., Klonisch T., de Souza J.B., Delves P.J., Lund T. (1997). Comparison of nucleic acid and protein immunization for induction of antibodies specific for HIV-1 gp120. Clin. Exp. Immunol..

[12] Jiao S.S., Williams P., Berg R.K., Hodgeman B.A., Liu L.J., Repetto G., Wolff J.A. (1992). Direct gene transfer into nonhuman primate myofibers *in vivo*. Hum. Gene Ther..

[13] Wang B., Boyer J., Srikantan V., Uqen K., Gilbert L., Phan C., Dang K., Merva M., Aqadjanyan M.G. (1995). Induction of humoral and cellular immune responses to the human immunodeficiency type 1 virus in nonhuman primates by *in vivo* DNA inoculation. Virology.

[14] Boyer J.D., Uqen K.E., Wang B., Aqadjanyan M., Gilbert L., Baqarazzi M.L., Chatterqoon M., Frost P., Javadian A., Williams W.V. (1997). Protection of chimpanzees from high-dose heterologous HIV-1 challenge by DNA vaccination. Nat. Med..

[15] Donnelly J.J., Ulmer J.B., Liu M.A. (1997). DNA vaccines. Annu. Rev. Immunol..

[16] Letvin N.L., Montefiori D.C., Yasutomi Y., Perry H.C., Davies M.E., Lekutis C., Alroy M., Freed D.C., Lord C.I., Handt L.K. (1997). Potent, protective anti-HIV immune responses generated by bimodal HIV envelope DNA plus protein vaccination. Proc. Natl. Acad. Sci. USA.

[17] Fuller D.H., Simpson L., Cole K.S., Clements J.E., Panicali D.L., Montelaro R.C., Murphey-Corb M., Haynes J.R. (1997). Gene gun-based nucleic acid immunization alone or in combination with recombinant vaccinia vectors suppresses virus burden in rhesus macaques challenged with a heterologous SIV. Immunol. Cell Biol..

[18] Schneider J., Gilbert S.C., Blanchard T.J., Hanke T., Robson K.J., Hannan C.M., Becker M., Sinden R., Smith G.L., Hill A.V. (1998). Enhanced immunogenicity for CD8+ T cell induction and complete protective efficacy of malaria DNA vaccination by boosting with modified vaccinia virus Ankara. Nat. Med..

[19] Robinson H.L., Montefiori D.C., Johnson P., Manson K.H., Kalish M., Lifson J.D., Rizvi T.A., Lu S., Hu S.L., Mazzara G.P. (1999). Neutralizing antibody-independent containment of immunodeficiency virus challenges by DNA priming and recombinant pox virus booster immunizations. Nat. Med..

[20] Allen T.M., Voqel T.U., Fuller D.H., Mothé B.R., Steffen S., Boyson J.E., Shipley T., Fuller J., Hanke T., Sette A. (2000). Induction of AIDS virus-specific CTL activity in fresh, unstimulated peripheral blood lymphocytes from rhesus macaques vaccinated with a DNA prime/modified vaccinia virus Ankara boost regimen. J. Immunol..

[21] Hanke T., McMichael A. (1999). Pre-clinical development of a multi-CTL epitope-based DNA prime MVA boost vaccine for AIDS. Immunol. Lett..

[22] Amara R.R., Villinger F., Altman J.D., Lydy S.L., O’Neil S.P., Staprans S.I., Montefiori D.C., Xu Y., Herndon J.G., Wyatt L.S. (2001). Control of a mucosal challenge and prevention of AIDS by a multiprotein DNA/MVA vaccine. Science.

[23] Mwau M., Cebere I., Sutton J., Chikoti P., Winstone N., Wee E.G., Beattie T., Chen Y.H., Dorrell L., McShane H. (2004). A human immunodeficiency virus 1 (HIV-1) clade A vaccine in clinical trials: Stimulation of HIV-specific T-cell responses by DNA and recombinant modified vaccinia virus Ankara (MVA) vaccines in humans. J. Gen. Virol..

[24] Mulligan M.J., Russell N.D., Celum C., Kahn J., Noonan E., Montefiori D.C., Ferrari G., Weinhold K.J., Smith J.M., Amara R.R. (2006). Excellent safety and tolerability of the human immunodeficiency virus type 1 pGA2/JS2 plasmid DNA priming vector vaccine in HIV type 1 uninfected adults. AIDS Res. Hum. Retrovir..

[25] Ondondo B.O., Yang H., Dong T., di Gleria K., Suttill A., Conlon C., Brown D., Williams P., Rowland-Jones S.L., Hanke T. (2006). Immunisation with recombinant modified vaccinia virus Ankara expressing HIV-1 gag in HIV-1-infected subjects stimulates broad functional CD4+ T cell responses. Eur. J. Immunol..

[26] Goonetilleke N., Moore S., Dally L., Winstone N., Cebere I., Mahmoud A., Pinheiro S., Gillespie G., Brown D., Loach V. (2006). Induction of multifunctional human immunodeficiency virus type 1 (HIV-1)-specific T cells capable of proliferation in healthy subjects by using a prime-boost regimen of DNA- and modified vaccinia virus Ankara-vectored vaccines expressing HIV-1 Gag coupled to CD8+ T-cell epitopes. J. Virol..

[27] Sandstrom E., Nilsson C., Hejdeman B., Brave A., Bratt G., Robb M., Cox J., Vancott T., Marovich M., Stout R. (2008). Broad immunogenicity of a multigene, multiclade HIV-1 DNA vaccine boosted with heterologous HIV-1 recombinant modified vaccinia virus Ankara. J. Infect Dis..

[28] Cebere I., Dorrell L., McShane H., Simmons A., McCormack S., Schmidt C., Smith C., Brooks M., Roberts J.E., Darwin S.C. (2006). Phase I clinical trial safety of DNA- and modified virus Ankara-vectored human immunodeficiency virus type 1 (HIV-1) vaccines administered alone and in a prime-boost regime to healthy HIV-1-uninfected volunteers. Vaccine.

[29] Hanke T., Goonetilleke N., McMichael A.J., Dorrell L. (2007). Clinical experience with plasmid DNA- and modified vaccinia virus Ankara-vectored human immunodeficiency virus type 1 clade A vaccine focusing on T-cell induction. J. Gen. Virol..

[30] Guimaraes-Walker A., Mackie N., McCormack S., Hanke T., Schmidt C., Gilmour J., Barin B., McMichael A., Weber J., Legg K. (2008). Lessons from IAVI-006, a phase I clinical trial to evaluate the safety and immunogenicity of the pTHr.HIVA DNA and MVA.HIVA vaccines in a prime-boost strategy to induce HIV-1 specific T-cell responses in healthy volunteers. Vaccine.

[31] Aboud S., Nilsson C., Karlen K., Marovich M., Wahren B., Sandstrom E., Gaines H., Biberfeld G., Godoy-Ramirez K. (2010). Strong HIV-specific CD4+ and CD8+ T-lymphocyte proliferative responses in healthy individuals immunized with an HIV-1 DNA vaccine and boosted with recombinant modified vaccinia virus ankara expressing HIV-1 genes. Clin. Vaccine Immunol..

[32] Goepfert P.A., Elizaga M.L., Sato A., Qin L., Cardinali M., Hay C.M., Hural J., DeRosa S.C., DeFawe O.D., Tomaras G.D. (2011). Phase 1 safety and immunogenicity testing of DNA and recombinant modified vaccinia Ankara vaccines expressing HIV-1 virus-like particles. J. Infect Dis..

[33] Nilsson C., Godoy-Ramirez K., Hejdeman B., Brave A., Gudmundsdotter L., Hallengard D., Currier J.R., Wieczorek L., Hasselrot K., Earl P.L. (2013). Broad and potent cellular and humoral immune responses after a second late HIV-modified vaccinia virus Ankara vaccination in HIV-DNA-primed and HIV-modified vaccinia virus Ankara-Boosted Swedish vaccinees. AIDS Res. Hum. Retrovir..

[34] Kutzler M.A., Weiner D.B. (2008). DNA vaccines: Ready for prime time?. Nat. Rev. Genet..

[35] Donnelly J.J., Liu M.A., Ulmer J.B. (2000). Antigen presentation and DNA vaccines. Am. J. Respir. Crit. Care Med..

[36] Corr M., Lee D.J., Carson D.A., Tighe H. (1996). Gene vaccination with naked plasmid DNA: Mechanism of CTL priming. J. Exp. Med..

[37] Asakura Y., Liu L.J., Shono N., Hinkula J., Kjerrstrom A., Aoki I., Okuda K., Wahren B., Fukushima J. (2000). Th1-biased immune responses induced by DNA-based immunizations are mediated via action on professional antigen-presenting cells to up-regulate IL-12 production. Clin. Exp. Immunol..

[38] Feltquate D.M., Heaney S., Webster R.G., Robinson H.L. (1997). Different T helper cell types and antibody isotypes generated by saline and gene gun DNA immunization. J. Immunol..

[39] Pillai V.B., Hellerstein M., Yu T., Amara R.R., Robinson H.L. (2008). Comparative studies on *in vitro* expression and *in vivo* immunogenicity of supercoiled and open circular forms of plasmid DNA vaccines. Vaccine.

[40] Flingai S., Czerwonko M., Goodman J., Kudchodkar S.B., Muthumani K., Weiner D.B. (2013). Synthetic DNA vaccines: Improved vaccine potency by electroporation and co-delivered genetic adjuvants. Front Immunol..

[41] MacGregor R.R., Ginsberg R., Ugen K.E., Baine Y., Kang C.U., Tu X.M., Higgins T., Weiner D.B., Boyer J.D. (2002). T-cell responses induced in normal volunteers immunized with a DNA-based vaccine containing HIV-1 env and rev. AIDS.

[42] Sardesai N.Y., Weiner D.B. (2011). Electroporation delivery of DNA vaccines: Prospects for success. Curr. Opin. Immunol..

[43] Luckay A., Sidhu M.K., Kjeken R., Megati S., Chong S.Y., Roopchand V., Garcia-Hand D., Abdullah R., Braun R., Montefiori D.C. (2007). Effect of plasmid DNA vaccine design and *in vivo* electroporation on the resulting vaccine-specific immune responses in rhesus macaques. J. Virol..

[44] Sutter G., Moss B. (1992). Nonreplicating vaccinia vector efficiently expresses recombinant genes. Proc. Natl. Acad. Sci. USA.

[45] Carroll M.W., Moss B. (1997). Host range and cytopathogenicity of the highly attenuated MVA strain of vaccinia virus: propagation and generation of recombinant viruses in a nonhuman mammalian cell line. Virology.

[46] Earl P.L., Americo J.L., Wyatt L.S., Eller L.A., Whitbeck J.C., Cohen G.H., Eisenberg R.J., Hartmann C.J., Jackson D.L., Kulesh D.A. (2004). Immunogenicity of a highly attenuated MVA smallpox vaccine and protection against monkeypox. Nature.

[47] Seth A., Ourmanov I., Kuroda M.J., Schmitz J.E., Carroll M.W., Wyatt L.S., Moss B., Forman M.A., Hirsch V.M., Letvin N.L. (1998). Recombinant modified vaccinia virus Ankara-simian immunodeficiency virus gag pol elicits cytotoxic T lymphocytes in rhesus monkeys detected by a major histocompatibility complex class I/peptide tetramer. Proc. Natl. Acad. Sci. USA.

[48] Sadagopal S., Amara R.R., Montefiori D.C., Wyatt L.S., Staprans S.I., Kozyr N.L., McClure H.M., Moss B., Robinson H.L. (2005). Signature for long-term vaccine-mediated control of a Simian and human immunodeficiency virus 89.6P challenge: Stable low-breadth and low-frequency T-cell response capable of coproducing gamma interferon and interleukin-2. J. Virol..

[49] Amara R.R., Smith J.M., Staprans S.I., Montefiori D.C., Villinger F., Altman J.D., O’Neil S.P., Kozyr N.L., Xu Y., Wyatt L.S. (2002). Critical role for Env as well as Gag-Pol in control of a simian-human immunodeficiency virus 89.6P challenge by a DNA prime/recombinant modified vaccinia virus Ankara vaccine. J. Virol..

[50] Sadagopal S., Amara R.R., Kannanganat S., Sharma S., Chennareddi L., Robinson H.L. (2008). Expansion and exhaustion of T-cell responses during mutational escape from long-term viral control in two DNA/modified vaccinia virus Ankara-vaccinated and simian-human immunodeficiency virus SHIV-89.6P-challenged macaques. J. Virol..

[51] Amara R.R., Ibegbu C., Villinger F., Montefiori D.C., Sharma S., Nigam P., Xu Y., McClure H.M., Robinson H.L. (2005). Studies using a viral challenge and CD8 T cell depletions on the roles of cellular and humoral immunity in the control of an SHIV-89.6P challenge in DNA/MVA-vaccinated macaques. Virology.

[52] Amara R.R., Villinger F., Staprans S.I., Altman J.D., Montefiori D.C., Kozyr N.L., Xu Y., Wyatt L.S., Earl P.L., Herndon J.G. (2002). Different patterns of immune responses but similar control of a simian-human immunodeficiency virus 89.6P mucosal challenge by modified vaccinia virus Ankara (MVA) and DNA/MVA vaccines. J. Virol..

[53] Lai L., Kwa S.F., Kozlowski P.A., Montefiori D.C., Nolen T.L., Hudgens M.G., Johnson W.E., Ferrari G., Hirsch V.M., Felber B.K. (2012). SIVmac239 MVA vaccine with and without a DNA prime, similar prevention of infection by a repeated dose SIVsmE660 challenge despite different immune responses. Vaccine.

[54] Geissler M., Gesien A., Tokushige K., Wands J.R. (1997). Enhancement of cellular and humoral immune responses to hepatitis C virus core protein using DNA-based vaccines augmented with cytokine-expressing plasmids. J. Immunol..

[55] Barouch D.H., Santra S., Schmitz J.E., Kuroda M.J., Fu T.M., Wagner W., Bilska M., Craiu A., Zheng X.X., Krivulka G.R. (2000). Control of viremia and prevention of clinical AIDS in rhesus monkeys by cytokine-augmented DNA vaccination. Science.

[56] Baden L.R., Blattner W.A., Morgan C., Huang Y., Defawe O.D., Sobieszczyk M.E., Kochar N., Tomaras G.D., McElrath M.J., Russell N. (2011). Timing of plasmid cytokine (IL-2/Ig) administration affects HIV-1 vaccine immunogenicity in HIV-seronegative subjects. J. Infect Dis..

[57] Morrissey P.J., Bressler L., Park L.S., Alpert A., Gillis S. (1987). Granulocyte-macrophage colony-stimulating factor augments the primary antibody response by enhancing the function of antigen-presenting cells. J. Immunol..

[58] Elliott M.J., Strasser A., Metcalf D. (1991). Selective up-regulation of macrophage function in granulocyte-macrophage colony-stimulating factor transgenic mice. J. Immunol..

[59] Haddad D., Ramprakash J., Sedegah M., Charoenvit Y., Baumgartner R., Kumar S., Hoffman S.L., Weiss W.R. (2000). Plasmid vaccine expressing granulocyte-macrophage colony-stimulating factor attracts infiltrates including immature dendritic cells into injected muscles. J. Immunol..

[60] Robinson H.L., Montefiori D.C., Villinger F., Robinson J.E., Sharma S., Wyatt L.S., Earl P.L., McClure H.M., Moss B., Amara R.R. (2006). Studies on GM-CSF DNA as an adjuvant for neutralizing Ab elicited by a DNA/MVA immunodeficiency virus vaccine. Virology.

[61] Lai L., Vodros D., Kozlowski P.A., Montefiori D.C., Wilson R.L., Akerstrom V.L., Chennareddi L., Yu T., Kannanganat S., Ofielu L. (2007). GM-CSF DNA: An adjuvant for higher avidity IgG, rectal IgA, and increased protection against the acute phase of a SHIV-89.6P challenge by a DNA/MVA immunodeficiency virus vaccine. Virology.

[62] Lai L., Kwa S., Kozlowski P.A., Montefiori D.C., Ferrari G., Johnson W.E., Hirsch V., Villinger F., Chennareddi L., Earl P.L. (2011). Prevention of infection by a granulocyte-macrophage colony-stimulating factor co-expressing DNA/modified vaccinia Ankara simian immunodeficiency virus vaccine. J. Infect Dis..

[63] Hellerstein M., Xu Y., Marino T., Lu S., Yi H., Wright E.R., Robinson H.L. (2012). Co-expression of HIV-1 virus-like particles and granulocyte-macrophage colony stimulating factor by GEO-D03 DNA vaccine. Hum. Vaccin. Immunother..

[64] Van Kooten C., Banchereau J. (2000). CD40-CD40 ligand. J. Leukoc. Biol..

[65] Van Mierlo G.J., den Boer A.T., Medema J.P., van der Voort E.I., Fransen M.F., Offringa R., Melief C.J., Toes R.E. (2002). CD40 stimulation leads to effective therapy of CD40(−) tumors through induction of strong systemic cytotoxic T lymphocyte immunity. Proc. Natl. Acad. Sci. USA.

[66] French R.R., Chan H.T., Tutt A.L., Glennie M.J. (1999). CD40 antibody evokes a cytotoxic T-cell response that eradicates lymphoma and bypasses T-cell help. Nat. Med..

[67] Quezada S.A., Jarvinen L.Z., Lind E.F., Noelle R.J. (2004). CD40/CD154 interactions at the interface of tolerance and immunity. Annu. Rev. Immunol..

[68] Kishi Y., Aiba Y., Higuchi T., Furukawa K., Tokuhisa T., Takemori T., Tsubata T. (2010). Augmented antibody response with premature germinal center regression in CD40L transgenic mice. J. Immunol..

[69] Crotty S. (2011). Follicular helper CD4 T cells (TFH). Annu. Rev. Immunol..

[70] Vonderheide R.H., Dutcher J.P., Anderson J.E., Eckhardt S.G., Stephans K.F., Razvillas B., Garl S., Butine M.D., Perry V.P., Armitage R.J. (2001). Phase I study of recombinant human CD40 ligand in cancer patients. J. Clin. Oncol..

[71] Stone G.W., Barzee S., Snarsky V., Kee K., Spina C.A., Yu X.F., Kornbluth R.S. (2006). Multimeric soluble CD40 ligand and GITR ligand as adjuvants for human immunodeficiency virus DNA vaccines. J. Virol..

[72] Gomez C.E., Najera J.L., Sanchez R., Jimenez V., Esteban M. (2009). Multimeric soluble CD40 ligand (sCD40L) efficiently enhances HIV specific cellular immune responses during DNA prime and boost with attenuated poxvirus vectors MVA and NYVAC expressing HIV antigens. Vaccine.

[73] Liu J., Yu Q., Stone G.W., Yue F.Y., Ngai N., Jones R.B., Kornbluth R.S., Ostrowski M.A. (2008). CD40L expressed from the canarypox vector, ALVAC, can boost immunogenicity of HIV-1 canarypox vaccine in mice and enhance the *in vitro* expansion of viral specific CD8+ T cell memory responses from HIV-1-infected and HIV-1-uninfected individuals. Vaccine.

[74] Wyzgol A., Yu Q., Stone G.W., Yue F.Y., Ngai N., Jones R.B., Kornbluth R.S., Ostrowski M.A. (2009). Trimer stabilization, oligomerization, and antibody-mediated cell surface immobilization improve the activity of soluble trimers of CD27L, CD40L, 41BBL, and glucocorticoid-induced TNF receptor ligand. J. Immunol..

[75] Calarota S.A., Leandersson A.C., Bratt G., Hinkula J., Klinman D.M., Weinhold K.J., Sandstrom E., Wahren B. (1999). Immune responses in asymptomatic HIV-1-infected patients after HIV-DNA immunization followed by highly active antiretroviral treatment. J. Immunol..

[76] Harari A., Bart P.A., Stohr W., Tapia G., Garcia M., Medjitna-Rais E., Burnet S., Cellerai C., Erlwein O., Barber T. (2008). An HIV-1 clade C DNA prime, NYVAC boost vaccine regimen induces reliable, polyfunctional, and long-lasting T cell responses. J. Exp. Med..

[77] Mehendale S., Thakar M., Sahay S., Kumar M., Shete A., Sathyamurthi P., Verma A., Kurle S., Shrotri A., Gilmour J. (2013). Safety and immunogenicity of DNA and MVA HIV-1 subtype C vaccine prime-boost regimens: a phase I randomised Trial in HIV-uninfected Indian volunteers. PLoS One.

[78] Hayes P., Gilmour J., von Lieven A., Gill D., Clark L., Kopycinski J., Cheeseman H., Chung A., Alter G., Dally L. (2013). Safety and immunogenicity of DNA prime and modified vaccinia ankara virus-HIV subtype C vaccine boost in healthy adults. Clin. Vaccine Immunol..

